# Preemptive *TPMT* Genotyping and Adherence to Genotype-Based Therapeutic Recommendations Reduces the Healthcare Cost in Patients Receiving Azathioprine or 6-Mercaptopurine for Autoimmune Diseases

**DOI:** 10.3390/jpm13081208

**Published:** 2023-07-29

**Authors:** Sarahí Valdez-Acosta, Pablo Zubiaur, Miguel Angel Casado, Jesús Novalbos, Ana Casajús, Diana Campodónico, Itziar Oyagüez, Francisco Abad-Santos

**Affiliations:** 1 Ethics Committee for Investigation with Medicinal Products (CEIm), Fundación de Investigación Biomédica (FIBH12O), Instituto de Investigación Sanitaria Hospital 12 de Octubre (imas12), 28041 Madrid, Spain; sarahivaldez.imas12@h12o.es; 2 Clinical Pharmacology Department, Hospital Universitario de La Princesa, Pharmacology Department of Faculty of Medicine, Universidad Autónoma de Madrid (UAM), Instituto de Investigación Sanitaria La Princesa (IP), 28006 Madrid, Spain; jesus.novalbos.externo@salud.madrid.org (J.N.); ana.casajus@salud.madrid.org (A.C.); diana.campodonico@salud.madrid.org (D.C.); 3 Pharmacoeconomics & Outcomes Research Iberia S.L. (PORIB), Pozuelo de Alarcón, 28224 Madrid, Spain; ma_casado@porib.com (M.A.C.); ioyaguez@porib.com (I.O.); 4 Centro de Investigación Biomédica en Red de Enfermedades Hepáticas y Digestivas (CIBERehd), Instituto de Salud Carlos III, 28006 Madrid, Spain

**Keywords:** TPMT, azathioprine, mercaptopurine, cost analysis

## Abstract

A cost analysis of thiopurine treatment was carried out in 257 patients, with 153 preemptively genotyped for *TPMT* and 104 retrospectively genotyped in a Spanish setting. The healthcare cost was significantly higher in patients retrospectively genotyped compared to those who were preemptively genotyped (*p* < 0.001). TPMT intermediate metabolizers (IMs) (n = 23) showed a 3.3-fold higher healthcare cost when compared to normal metabolizers (NMs) (*p* < 0.001). The healthcare cost in patients with a TPMT IM phenotype whose physician adhered to the genotype-informed recommendation was similar than the cost in TPMT NMs and was significantly lower than IMs whose physician did not adhere to the therapeutic recommendation (3.8-fold, *p* = 0.016). Myelotoxicity occurrence was significantly lower in patients preemptively vs. retrospectively genotyped (2.0% and 21.2%, respectively, *p* < 0.001). Patients who developed myelotoxicity showed a significantly higher healthcare cost than those who did not (4.10-fold, *p* < 0.001). Overall, 87% of patients whose dose was not adjusted despite being TPMT IMs suffered myelotoxicity, while only one of the eight patients (13%) whose dose was adjusted suffered myelotoxicity (*p* < 0.001). In conclusion, *TPMT* preemptive genotyping and physician adherence to genotype-informed therapeutic recommendations prevents myelotoxicity and significantly reduces the healthcare cost, and it is therefore essential for the sustainability of the Spanish healthcare system.

## 1. Introduction

Thiopurines are drugs that are widely used in the treatment of autoimmune diseases such as inflammatory bowel disease, rheumatoid arthritis, multiple sclerosis, or systemic lupus erythematosus. They are also indicated for the treatment of different types of leukemias. Thiopurines are antimetabolite purine analogs, fundamental precursors of DNA and RNA, and are essential for cell division and growth. In Spain, 6-mercaptopurine and azathioprine are marketed; thioguanine is also approved but is not currently marketed. Azathioprine is a prodrug of 6-mercaptopurine [1]. After administration, azathioprine is metabolized to 6-mercaptopurine by glutathione-S transferase (GST). 6-mercaptopurine is extensively metabolized by thiopurine methyltransferase (TPMT) to inactive metabolites [1]. The active metabolites are responsible for the therapeutic effect of the drug and its toxicity. Likewise, NUDT15 encodes for the enzyme nudix hydrolase 15, which transforms active intermediate metabolites into inactive ones. The most worrisome adverse reaction that thiopurines can produce is bone marrow suppression. Therefore, it was traditionally recommended to initiate treatment at low doses and adjust the dose gradually based on the leukocyte count [1].

In 2011, the Clinical Pharmacogenetics Implementation Consortium (CPIC) published its guideline on thiopurine dosing based on TPMT genotype-informed phenotype [2]. This guideline recommended reducing the starting dose for TPMT intermediate metabolizers (IMs) to 30–70% of the standard dose and by 10-fold and dose thrice weekly to poor metabolizers (PMs) or selecting an alternative drug depending on the indication. In 2019, an update of the guideline was published, where the same recommendations for NUDT15 IMs and PMs, respectively, were included [3].

Despite the positive effect of pharmacogenetic biomarkers on clinical practice, numerous barriers still obstruct the implementation of clinical pharmacogenetics, including the uncertainty of its economic impact cost-effectiveness. However, in recent years, numerous cost-effectiveness analyses have been carried out. In the case of *TPMT* prospective genotyping, its cost-effectiveness was analyzed in various studies [4,5,6]. Although some of these works strongly argue in favor of the implementation of preemptive genotyping, each country, healthcare system, and hospital administration are unique. Additional complex variables may condition the decision to implement these procedures, for instance, the healthcare costs associated with drug toxicity (e.g., intensive care unit or emergency room admissions), the public or private nature of the system, the regulatory requirements associated with the tests, equipment, and qualified healthcare personnel to perform the tests, etc. This implies that, although the individual benefit of preemptive genotyping may be proven, the collective benefit must be carefully assessed according to the different local or national idiosyncrasies.

To our knowledge, no cost analysis of the thiopurine treatment related to *TPMT* genotype has been published for the Spanish healthcare system. In a recent study [1], we demonstrated that *TPMT* preemptive genotyping significantly reduced the incidence of leukopenia and other adverse drug reactions (ADRs) in TPMT intermediate metabolizers (IMs) to the level of normal metabolizers (NMs), in the patients preemptively genotyped at our center (Hospital Universitario de La Princesa, Madrid, Spain) receiving azathioprine. Our next aim, which is the purpose of this work, was to perform a cost analysis of thiopurine prescription in a cohort of patients with *TPMT* preemptive genotyping compared to another cohort of patients that were genotyped after the beginning of treatment. This work is part of the La Princesa Multidisciplinary Initiative for the Implementation of Pharmacogenetics (PriME-PGx) [7].

## 2. Materials and Methods

### 2.1. Study Design and Population

The present work was designed as an observational, descriptive, and retrospective study analyzing treatment costs in a cohort of patients diagnosed with an autoimmune disease, prescribed azathioprine or *6*-mercaptopurine, and genotyped for *TPMT*. For the inclusion of patients, the database of *TPMT* determinations from the Pharmacogenetics Unit, Clinical Pharmacology Department, Hospital Universitario de La Princesa (SFC-HUP) was reviewed for the 2009–2016 period; CPIC guideline recommendations were followed during this period and are still implemented nowadays. The inclusion criteria comprised: patients ≥ 18 years old, with an autoimmune disease diagnosis, genotyped for *TPMT* at the Pharmacogenetics Unit of SFC-HUP and treated with azathioprine or mercaptopurine. The exclusion criteria included pediatric, hematologic, and oncologic patients, or patients derived from other hospitals. The study was approved by the Independent Ethics Board (IEB) of Hospital Universitario de La Princesa, Madrid Spain, with the registration number 3163. In compliance with the Spanish Biomedical Law and the Revised Declaration of Helsinki, the IEB approved the waiver of collecting informed consent again from the patients, since they had already consented previously to their routine practitioner, and it was research of public interest, without negatively affecting the patient’s interests, and was carried out in a completely anonymized manner. The follow-up period was 6 months after the initiation of thiopurine pharmacotherapy, regardless of the chronology of genotyping. Patients’ medical records were reviewed, and the clinical variables were collected, including demographic variables such as age, sex, weight, smoking habit, disease diagnosis (e.g., Crohn’s disease, atopic dermatitis, myasthenia gravis, etc.), information regarding azathioprine or mercaptopurine prescription (e.g., dose, treatment duration, etc.), and concomitant medications.

### 2.2. Genotyping

The *TPMT* genotype and TPMT genotype-informed phenotype was retrieved from the Pharmacogenetics Unit database. The genotyping strategy comprised, for the 2006–2010 period, the Sanger sequencing of the entire gene. For the 2009–2016 period, it comprised the core variants of TPMT*2 (rs1800462), *3A (rs1800460 and rs1142345), *3B (rs1800460), and *3C (rs1142345), as described in the PharmGKB Gene-specific Information Tables for *TPMT* website (available at https://www.pharmgkb.org/page/tpmtRefMaterials, accessed on 28 June 2023), in accordance with the Clinical Pharmacogenetics Implementation Consortium (CPIC)’s 2018 update on the guideline for thiopurine dosing based on *TPMT* genotyping [2]. The absence of any variant was defaulted as TPMT*1. Variants were genotyped with a LightCycler 2.0 instrument, as indicated previously (Roche Diagnostics, Mannheim, Germany).

### 2.3. Cost Analysis

Genotype, demographic, and disease information were used as independent variables; the main dependent variable was considered the total cost of treatment. A national database from the Oblikue consulting company was used to determine the unit cost of each clinical test, costs per admission, consultation with services, etc., associated with patient management (available at: http://esalud.oblikue.com/, accessed on 1 September 2017). The Supplementary Table S1 depicts all the individual costs per item, including lab tests and procedures, image studies, surgical procedures, consultations, and admissions per Hospital Department. This information was used to calculate the total healthcare cost per patient. The cost of *TPMT* genotyping was also retrieved from this source for homogenization (i.e., €41.68 per patient) instead of using the real cost at the Pharmacogenetics Unit of SFC-HUP.

### 2.4. Statistical Analysis

The statistical analysis was carried out using the software SPSS version 19 (IBM Corporation, Armonk, NY, USA). A descriptive analysis was made of the demographic and clinical characteristics of the patients. For categorical variables, percentages were provided and Chi-squared or Fisher exact-tests were used when appropriate. For the healthcare cost variable, a Shapiro–Wilk test was used to explore variable normality. For the comparison of treatment cost according to variables with two or three or more categories, the Mann–Whitney and Kruskal–Wallis nonparametric tests were used and the median and quartiles 1 and 3 were provided. The healthcare cost and individual costs of several sections (e.g., treatment cost or hospital admission cost) were analyzed according to the chronology of genotyping (i.e., preemptive vs. after initiating thiopurine treatment). Due to the high number of outliers, the mean and standard deviation were also provided. A *p* value lower than 0.05 was considered nominally significant. For pairwise comparisons after the Kruskal–Wallis test, the significance value was adjusted by the Bonferroni correction for multiple tests (i.e., a corrected *p* value is provided).

## 3. Results

A total of 485 requests were screened. After removing duplicates and excluding patients who did not meet the inclusion criteria, 257 patients were included, of which 153 (59.9%) had been preemptively genotyped and 104 (40.1%) had been retrospectively genotyped, or after initiating thiopurine treatment. The mean age was 53.4 ± 17.2 years old, 163 patients were female (63.4%), and the mean weight was 68.6 ± 14.4 kg. Patients preemptively genotyped were significantly older than those genotyped after initiating thiopurine treatment (56.2 ± 17.76 vs. 49.3 ± 17.8 years old, *p* < 0.001) but the weight was similar (70.0 ± 14.5 vs. 66.5 ± 13.2 kg, *p* = 0.109). Patients retrospectively genotyped showed a significantly higher number of TPMT IMs than patients preemptively genotyped (Table 1). TPMT*2 and *3A allele prevalence (1.6% and 3.9%) were significantly higher in the study population than in Europeans (0.2% and 3.4%, respectively) (*p* = 0.005 and *p* = 0.030, respectively); European prevalence data were obtained from Gene-Specific Information Tables available at https://www.pharmgkb.org/page/tpmtRefMaterials). No differences in phenotype prevalence were observed, with 91% NMs, 8% IMs, 0.2% PMs, and <1% of indeterminate or possible IMs in Europeans compared to 95.4% of NMs and 4.6% IMs observed here, *p* > 0.050.

Mean healthcare cost was €3013.65 ± 4201.68, with a median of €1550.76 and with a first and third quartile of €1011.63 and €2483.72 (Table 2), respectively, with a minimum cost of €180.93 and a maximum cost of €29,072.95. Expectedly, the distribution of the cost variable was asymmetrical (skewness value: 3.25) and leptokurtic, with long heavy tails (excess kurtosis: 12.54) (Supplementary Figure S1). The mean and SD cost values are shown throughout the results section to better reflect the real cost per patient for each independent variable. Total healthcare cost, cost of consultations, cost of hospitalization, and cost of concomitant medications were higher in patients genotyped after initiating thiopurine treatment (i.e., retrospectively genotyped) compared to patients who were preemptively genotyped (*p* < 0.05) (Table 2).

The Supplementary Table S2 shows the calculated total healthcare cost per patient and breakdown by cost of tests, consultations, hospital admissions, and cost of medication. The healthcare cost according to the baseline characteristics is shown in Table 3. Treatment with 6-mercaptopurine was related to a significantly higher healthcare cost compared to azathioprine (*p* = 0.044). Smokers were related to a significantly higher healthcare cost compared to former smokers (*p* value after the Bonferroni correction for multiple comparisons = 0.023) and to nonsmokers nominally (nominal *p* = 0.031). The healthcare cost was higher in patients with Crohn’s disease and ulcerative colitis compared to patients with pemphigus (corrected *p* = 0.002 and *p* = 0.028, respectively). Patients concomitantly treated with monoclonal antibodies showed a significantly higher healthcare cost than patients treated with any other drug (corrected *p* < 0.05 for all comparisons), and those treated with corticosteroids showed a higher healthcare cost than those concomitantly treated with mesalazine (*p* = 0.029). Patient’s sex or concomitant diseases had no impact on the healthcare cost.

TPMT IMs (n = 23) showed a 3.3-fold higher mean cost when compared to TPMT NMs (n = 234) (Table 3); when comparing the medians, differences were 4.7-fold (*p* < 0.001) (Table 4). Among the 23 TPMT IMs, physicians complied with the therapeutic recommendations issued by the Pharmacogenetics Unit for eight patients, while they did not for fifteen patients (Table 4). The healthcare cost in patients with a TPMT IM phenotype whose physicians adhered to the therapeutic recommendation was similar or slightly higher than the cost in TPMT NMs (€2960.22 ± 2313.86 and €2494.88 ± 3227.78; median: €1478.85 and €1919.95, respectively, *p* = 0.065), but significantly lower (3.8-fold) than IMs whose physician did not adhere to the therapeutic recommendation (€11,134.85 ± 8460.54; median €6706.63, *p* = 0.016) (Table 4). Moreover, 56% of TPMT IMs developed myelotoxicity (14 of 23) compared to 4.7% of TPMT NMs (11 of 234, *p* < 0.001). The prevalence of myelotoxicity was significantly lower in patients who were preemptively vs. retrospectively genotyped (2.0%, 3 of 153 and 21.2%, 22 of 104, *p* < 0.001). Patients who developed myelotoxicity showed a significantly higher cost of treatment (4.10-fold) than those who did not (*p* < 0.001) (Table 4). Overall, 13 of the 15 patients (87%) whose dose was not adjusted despite being TPMT IMs suffered myelotoxicity, while only 1 of the 8 patients (13%) whose dose was adjusted suffered myelotoxicity, as the prescriber complied with the genotype-informed recommendations (*p* < 0.001).

## 4. Discussion

Recently, we demonstrated that the genotype-guided prescription of azathioprine reduces the incidence of adverse drug reactions in TPMT IMs to a similar incidence as NMs [1] . The evidence that preemptive genotyping of *TPMT* prevents severe thiopurine ADRs is definitive. However, when considering the sustainability of healthcare systems, certain tests can be considered too costly despite being good value for money or beneficial for the patients. The lack of robust data demonstrating that these tests reduce healthcare costs in a particular location or population may lead hospital management to not include these tests in their portfolio of services.

Several studies have examined the cost-effectiveness of genotyping *TPMT* (thiopurine S-methyltransferase) in different populations and clinical contexts. Marra et al., 2002 [8], first suggested that *TPMT* genotyping could be valuable in specific clinical scenarios, although specific evidence was not provided. One year later, Oh et al., 2003 [9], first demonstrated the cost-effectiveness of *TPMT* genotyping in Korean patients undergoing azathioprine treatment. In 2005, Dubinsky et al. [10] focused on patients with Crohn’s disease treated with mercaptopurine or azathioprine. Their research showcased the cost-effectiveness of genotyping in this specific population, emphasizing its potential to guide treatment decisions and reduce healthcare expenses. Another study from 2006, focusing on acute lymphoblastic leukemia (ALL) patients, demonstrated the cost-effectiveness of *TPMT* genotyping in this particular malignancy [6]. The only cost-effectiveness study published to date on *TPMT* specific to the Spanish population dates back to 2009 [11], which also discussed the impact on the UK population. Although the discussion revolved around the potential benefits, conclusive evidence supporting the cost-effectiveness of genotyping in these countries was not established. In 2010, researchers concluded that genotyping for *TPMT* in idiopathic pulmonary fibrosis (IPF) could be cost-effective if the incidence of intermediate or poor metabolizers exceeded 12.5% [4]. However, in a study conducted in 2011, researchers suggested that *TPMT* genotyping might not be cost-effective in pediatric patients with ALL [12]. In 2014, a study demonstrated that *TPMT* genotyping reduced costs but potentially had a small negative impact on patient health [13]. This finding indicated the need for careful consideration of the potential benefits and drawbacks associated with genotyping. In 2019, *TPMT* genotyping and phenotyping was considered cost-effective in a French population prescribed with azathioprine [5]. Moreover, Sluiter et al., 2019, demonstrated the cost-effectiveness of *TPMT* genotyping in patients with inflammatory bowel disease (IBD) and demonstrated that the quality of life of IMs or PMs compared to NMs was similar after therapy adjustment [14]. Furthermore, in 2021, the genotyping of *TPMT* and *NUDT15* (nucleoside diphosphate-linked moiety X-type motif 15) in the Chinese population was found to be cost-effective [15]. Indeed, the inclusion of both genes was superior in terms of cost-effectiveness than *TPMT* genotyping alone. In contrast, in another study, NUDT15 genotyping was considered cost-effective only in Asians but “unrealistic” in Europeans (Caucasians) [16] because of the low frequency of no function alleles. In the latter study, published in 2020, *TPMT* genotyping was considered cost-effective for both populations, as it was able to avoid 43 severe myelosuppression cases per 10,000 patients in Europeans compared to 3.6 in Asians. Finally, a study in 2022 estimated the potential cost savings associated with *TPMT* genotyping in a Dutch population of 150,000 individuals [17]. Although the study highlighted the potential economic benefits, it did not provide conclusive evidence regarding the cost-effectiveness of genotyping in this specific population. In summary, there appears to be definitive evidence that prospective genotyping of *TPMT* offers improved patient quality of life and reduced healthcare costs. Every country presents unique idiosyncrasies and political contexts, and this influences how healthcare systems are financed. Therefore, in this work, we aimed to demonstrate the cost-effectiveness of *TPMT* genotyping in the Spanish context. To our knowledge, this is the first work that accurately describes the healthcare costs associated with thiopurine therapy in a Spanish public hospital. This was an observational study with real patients and real calculations of healthcare costs, not estimates. As many of these costs are common to other clinical settings, the Supplementary Material is of great value, as future pharmacoeconomic studies could be informed by this material.

In this work, patients treated with 6-mercaptopurine showed a significantly higher cost of treatment compared to azathioprine. In Spain, the cost of azathioprine 50 mg, 50 tablets is €10.40, and the cost of 6-mercaptopurine 50 mg/25 tablets is €15.30. The standard azathioprine dose is 2–3 mg/kg/day and 1.5 mg/kg/day for mercaptopurine. Assuming a patient’s weight of 70 kg, the daily treatment cost is €0.74 and €1.29, respectively. Azathioprine is generally the immunosuppressant of choice, but if azathioprine treatment fails, mercaptopurine may be tolerated [18]. As we did not observe differences in the incidence of myelotoxicity between patients treated with these two drugs, the higher cost associated with mercaptopurine may be related to the fact that these patients receive a second-line drug when there has already been an initial failure to azathioprine, and they are in a worse clinical situation than those who are prescribed first-line azathioprine.

Smokers were related to a significantly higher healthcare cost compared to former smokers and to nonsmokers, which is consistent with the higher morbidity and mortality associated with smoking and with a worse disease control. Furthermore, differences in cost were only observed between patients with pemphigus (lower healthcare cost) and patients with Crohn’s disease and ulcerative colitis (higher cost). However, the absence of additional associations is explained by the limited sample size within each disease group. With larger sample sizes, it would be expected to generally observe higher healthcare costs in patients with more severe pathologies. For instance, patients with systemic lupus erythematosus showed the highest cost (n = 12; €5817.94) and those with scleroderma showed the lowest (n = 3; €984.53), but these differences did not reach the statistical threshold for significance due to the low sample sizes. The same was observed when comparing the cost according to concomitant treatments. A relevant association observed here was the significantly higher cost associated with the concomitant treatment with monoclonal antibodies compared to the rest of patients. This is explained by two reasons: a) the evident higher cost of biologic treatments and b) the indication of this family of drugs being a surrogate marker of a worse clinical condition and, therefore, of greater morbidity, associated with higher healthcare costs.

The group of retrospectively genotyped patients was enriched in patients who had developed severe toxicity, which explained the higher healthcare cost associated with this group. The 3.3-fold higher mean cost was observed in TPMT IMs compared to TPMT NMs (i.e., a higher healthcare cost of more than €5700), and the cost of TPMT genotyping (less than €100) suggests that this test is cost-effective. However, it could be argued that this comparison is not appropriate, as the cost in IMs is higher than expected because some of them were genotyped retrospectively after the onset of severe toxicity, and not preventively. To demonstrate the impact of preemptive genotyping on the healthcare cost, we analyzed the cost of TPMT IMs who were preemptively genotyped and whose physician adhered to therapeutic recommendations and compared it to those whose physician did not adhere to therapeutic recommendations, or who were retrospectively genotyped. Patients who developed myelotoxicity showed a significantly higher healthcare cost than those who did not; physician adherence to genotyping-based therapeutic recommendations would have prevented myelotoxicity in up to 13 patients and significantly reduced the healthcare cost. This confirms not only that *TPMT* genotyping is cost-effective, but also that prescriber adherence is essential for the sustainability of the healthcare system.

In this work, the total treatment cost was comprehensively calculated by addressing several individual cost items, i.e., lab tests and procedures, image studies, surgical procedures, consultations, and admissions per Hospital Department. This controls all sources of increased health care costs. For example, a patient with severe toxicity may require hospital admission, and additional laboratory tests during admission, but also procedures specific to their morbidity. The personalized and itemized calculation of the health care cost per patient makes it possible to estimate the total cost per patient with great accuracy. This explains the high statistical power, the observed statistical significance, and the concluded clinical significance.

Interestingly, in our hospital, some physicians use *TPMT* genotyping for the prescription of thiopurines in the management of patients with autoimmune diseases, but others do not, arguing that they do not consider this biomarker useful for the selection and titration of thiopurine doses. Hopefully, the data provided in this manuscript will help to convince them and standardize the use of this biomarker in clinical practice.

As pharmacogenetic recommendations are not yet fully standardized, there is some confusion on the part of clinicians as to which recommendations should be followed; institutions such as CPIC or the Dutch Pharmacogenetics Working Group (DPWG) issue recommendations do not always coincide, and this leads to indecision on the part of the clinician. For instance, CPIC recommends a 30–70% of the standard dose for TPMT IMs while DPWG recommends a 50% reduction. In this work, which is not a randomized clinical trial with a strict and standardized application of the genotype-informed recommendations, the pragmatic use of these biomarkers has been shown to reduce healthcare costs and improve treatment tolerability. In our opinion, this is a relief, as it indicates that what is important is not the exact recommendation of the most appropriate dose, but the intention to protect and monitor patients with risk genotypes.

### Limitations

This study has some limitations. Concerning the study design, a fully prospective design evaluating the impacts of preemptive genotyping would have been a superior design to demonstrate the impact of prospective genotyping on healthcare costs. Nevertheless, our cohort of 153 patients was sufficient to demonstrate that *TPMT* preemptive genotyping significantly reduces healthcare costs and the importance of prescriber adherence to therapeutic recommendations, while valuable information on another retrospective genotyping cohort of 104 patients is provided. Second, the cost-effectiveness of *NUDT15* genotyping was not analyzed, another biomarker with robust evidence whose genotyping reduces ADR incidence and associated healthcare costs. However, given the low frequency of non-functional variants (i.e., no function variants, or variants coding for an inactive or almost completely inactive TPMT enzyme) in Spain, it would have required a huge sample size, and this study cohort would most likely fail to demonstrate cost-effectiveness due to the limited statistical power.

## 5. Conclusions

*TPMT* preemptive genotyping demonstrated a cost-saving strategy in a Spanish population. Physician adherence to genotype-based therapeutic recommendations prevents myelotoxicity and significantly reduces the healthcare cost.

## Figures and Tables

**Table 1:** TPMT phenotype according to the chronology of genotyping.

TPMT Phenotype	Genotypes	Patients Preemptively Genotyped (n = 153)	Patients Retrospectively Genotyped (n = 104)	*p* Value
NM (n = 234, 91.1%)	*1/*1	146 (95.4%)	88 (84.6%)	0.006
IM (n = 23, 8.9%)	*1/*3A, n = 17; *1/*2, n = 4; *1/*3C, n = 1; *1/*19, n = 1	7 (4.6%)	16 (15.4%)	

The absence of any variant was defaulted TPMT*1.

**Table 2:** Breakdown of the different cost items according to the chronology of genotyping.

Variable	n	Mean (€)	SD	Median (€)	Q1	Q3	*p* Value
Total healthcare cost	257	3013.65	4201.68	1550.76	1011.63	2483.72	
Preemptive	153	2071.89	2721.38	1250.49	916.89	1863.38	<0.001
Retrospective	104	4399.11	5449.17	2126.79	1275.76	6300.79	
Cost of lab and image tests	257	513.57	481.13	366.42	205.69	1073.69	
Preemptive	153	462.34	431.80	358.76	211.01	554.24	0.295
Retrospective	104	588.95	539.03	391.73	198.3	766.21	
Cost of consultations	245	859.75	681.48	749.51	446.69	1108.02	
Preemptive	153	732.02	710.17	604.51	371.53	938.47	<0.001
Retrospective	92	1047.66	591.71	1007.51	600.35	1340.43	
Cost of hospitalization	92	3242.90	5516.63	278.37	104.52	4668.89	
Preemptive	46	1804.63	3501.94	209.05	104.52	1448.04	0.015
Retrospective	46	4681.18	6711.92	1167.73	104.52	6983.76	
Cost of thiopurine treatment	257	80.57	45.20	81.18	45.10	99.22	
Preemptive	153	77.07	39.05	72.16	45.10	99.22	0.332
Retrospective	104	85.72	52.75	81.18	45.10	99.22	
Cost of concomitant treatment	221	463.84	1558.94	16.94	15.00	69.44	
Preemptive	136	290.14	1118.36	15.00	11.90	30.00	0.023
Retrospective	85	741.77	2056.20	20.00	15.00	326.84	

**Table 3:** Healthcare cost according to baseline characteristics.

Variable	n	Mean (€)	SD	Median (€)	Q1	Q3	*p* Value
**Total**	257	3013.65	4201.68	1550.76	1011.63	2483.72	N/A
**Treatment**							
Azathioprine	242	2914.58	4136.24	1545.61	981.01	2344.84	0.044
6-mercaptopurine	15	4611.91	5039.87	2391.19	1300.60	7010.61	
**Sex**							
Male	94	2850.12	4088.05	1586.60	980.24	3177.07	0.953
Female	163	3107.95	4275.43	1580.92	920.36	2357.11	
**Smoking**							
Nonsmoker	134	2899.68	4089.74	1512.33	1020.89	2382.82	0.020 (Smoker vs. Former smoker, *p* = 0.023)
Smoker	63	3939.09	5140.52	1945.98	1250.49	3299.48	
Former smoker	50	2392.66	3348.00	1421.39	868.70	2020.90	
**Disease**							
Crohn’s disease	51	4805.60	5741.63	1988.24	1300.60	6706.63	<0.001 (Crohn’s disease vs. Pemphigus, *p* = 0.002; Ulcerative Colitis vs. Pemphigus, *p* = 0.028)
Pemphigus	28	1743.44	2650.15	980.76	740.46	1666.38	
Ulcerative Colitis	21	3349.91	3316.88	2130.31	1402.60	3177.33	
Diffuse interstitial lung disease	20	2664.12	3481.57	1415.95	1173.92	1840.56	
Vasculitis	17	1709.89	2438.22	1056.83	746.56	1644.11	
Systemic lupus erythematosus	12	5817.94	9639.15	998.41	713.57	8785.86	
Sjögren’s Syndrome	10	2134.70	1326.67	2017.71	1385.53	2351.94	
Atopic dermatitis	6	1014.94	311.88	1047.72	706.46	1231.50	
Myasthenia gravis	5	1747.91	337.93	1866.43	1500.12	1936.45	
Autoimmune hepatitis	4	3207.41	3940.03	1416.71	983.45	7222.06	
Optic neuritis	3	1217.96	523.36	1319.28	651.34		
Dermatomyositis	4	2717.48	2313.85	2168.56	849.32	5134.56	
Immune Thrombocytopenic Purpura	3	1492.03	795.75	1849.56	580.22		
Mixed connective tissue disease	3	1412.52	938.37	1552.20	412.13		
Multiple sclerosis	3	1046.58	270.67	1012.77	794.40		
Scleroderma	3	984.53	101.54	938.43	914.22		
Other	14	2050.31	2602.44	1161.04	738.74	2158.32	
**Concomitant medications**							
Corticosteroids	155	2307.49	3430.12	1264.20	850.15	2048.65	<0.001 (Monoclonal antibodies vs. all, *p* < 0.05; corticosteroids vs. mesalazine, *p* = 0.029)
Mesalazine	28	2701.70	2301.62	1889.60	1373.42	2783.21	
Monoclonal antibodies	15	8848.14	3217.17	7658.16	6600.64	10,052.44	
Omeprazole	7	4227.22	7519.16	1332.57	1207.01	1945.98	
Other	16	3047.65	3333.10	1647.40	938.52	3268.88	
**Concomitant diseases**							
Hypertension	42	3029.53	4181.73	1631.23	885.07	3124.48	0.107
Other autoimmune diseases	32	4458.37	5779.56	2048.44	1385.64	5856.96	
Neurological diseases	12	1460.71	976.49	1210.60	751.74	1770.40	
Dyslipidemia	9	1211.71	619.32	1164.62	642.67	1694.97	
Diabetes	9	4085.73	5394.11	1677.13	992.08	7367.45	
Asthma	7	1479.05	562.96	1239.45	1043.10	2046.31	
Cancer	7	3533.14	4374.87	1595.91	1232.50	4563.01	
Hepatitis	4	5095.51	5975.92	2424.37	1575.35	11,286.81	
Others	18	2283.12	2098.94	1431.05	960.78	2601.50	

**Table 4:** Healthcare cost according to TPMT phenotype, chronology of genotype request, and compliance with therapeutic recommendations based on the genotype-informed phenotype and development of myelotoxicity.

Variable	n	Mean (€)	SD	Median (€)	Q1	Q3	*p* Value
**Total**	257	3013.65	4201.68	1550.76	1011.63	2483.72	N/A
**Genotype request**							
Retrospective	104	4399.11	5449.17	2126.79	1275.76	6300.79	<0.001
Preemptive	153	2071.89	2721.38	1250.49	916.89	1863.38	
**TPMT Phenotype**							
NM (*1/*1)	234	2494.88	3227.78	1478.85	973.78	2252.51	<0.001
IM (*1/*3A, n = 17; *1/*2, n = 4; *1/*3C, n = 1; *1/*19, n = 1)	23	8291.50	7943.72	7010.61	1671.66	10,965.94	
**Compliance with dose adjustment recommendation for TPMT intermediate metabolizers (n = 23)**							
Physician complied	8	2960.22	2313.86	1919.95	1607.87	5134.56	0.016
Physician did not comply	15	11,134.86	8460.54	9368.92	6706.63	13,915.33	
**Developed myelotoxicity**							
No	232	2317.37	2905.00	1455.69	971.91	2110.14	<0.001
Yes	25	9475.06	7675.22	7658.16	3003.29	11,190.77	

## Data Availability

All data are already available in the manuscript and Supplementary Materials.

## Supplementary Materials

The following supporting information can be downloaded at: https://www.mdpi.com/article/10.3390/jpm13081208/s1, Table S1: Individual cost per item, including lab tests and procedures, image studies, surgical procedures, consultations, and admissions per Hospital Department; Table S2: Total healthcare cost per patient and breakdown by cost of tests, consultations, hospital admissions and cost of medication; Figure S1: Normality plots for the “Cost per patient (€)” variable. (a) Histogram showing the asymmetrical, leptokurtic distribution of the variable, (b) Quantile-quantile plot of the observed “Cost per patient (€)” value and the expected normal value.
