# Supplementary material for: Preemptive TPMT Genotyping and Adherence to Genotype-Based Therapeutic Recommendations Reduces the Healthcare Cost in Patients Receiving Azathioprine or 6-Mercaptopurine for Autoimmune Diseases
Source: J Pers Med. 2023 Jul 29;13(8):1208. doi: 10.3390/jpm13081208 (PMC10455787; doi:10.3390/jpm13081208)
Supplement: Supplementary file 1 [file jpm-13-01208-s001.zip › jpm-2507515-supplementary.pdf]

Supplementary Material: Preemptive *TPMT* Genotyping and Adherence to Genotype-Based Therapeutic Recommendations Reduces the Healthcare Cost in Patients Receiving Azathioprine or 6-Mercaptopurine for Autoimmune Diseases

Sarahí Valdez-Acosta [1,†], Pablo Zubiaur [2,*,†], Miguel Angel Casado [3], Jesús Novalbos [2], Ana Casajús [2], Diana Campodónico [2], Itziar Oyagüez [3] and Francisco Abad-Santos [2,4,*]

[1] Ethics Committee for Investigation with Medicinal Products (CEIm), Fundación de Investigación Biomédica (FIBH12O), Instituto de Investigación Sanitaria Hospital 12 de Octubre (imas12), Madrid, Spain, 28041; sarahivaldez.imas12@h12o.es

[2] Clinical Pharmacology Department, Hospital Universitario de La Princesa, Pharmacology Department of Faculty of Medicine, Universidad Autónoma de Madrid (UAM), Instituto de Investigación Sanitaria La Princesa (IP), Madrid, Spain, 28006; jesus.novalbos.externo@salud.madrid.org (J.N.); ana.casajus@salud.madrid.org (A.C.); diana.campodonico@salud.madrid.org (D.C.)

[3] Pharmacoeconomics & Outcomes Research Iberia S.L. (PORIB), Pozuelo de Alarcón, Madrid, Spain, 28224; ma_casado@porib.com (M.A.C.); ioyaguez@porib.com (I.O.)

[4] Centro de Investigación Biomédica en Red de Enfermedades Hepáticas y Digestivas (CIBERehd), Instituto de Salud Carlos III, Madrid, Spain28006

Supplementary Table S1. Individual cost per item, including lab tests and procedures, image studies, surgical procedures, consultations, and admissions per Hospital Department.

**Item**	**Cost (€)**
Blood count	4.29
Biochemistry	31.63
Coagulation study	10.47
Urine analysis	8.44
Arterial blood gas analysis	4.25
Venous blood gas analysis	4.25
**Autoimmunity study**	
Proteinogram	9.04
Antinuclear antibodies (ANA)	29.52
Antineutrophil cytoplasmic antibodies (ANCA)	25.97
Anti-desmoglein antibodies	44.08
Complement C3-C4 proteins	12.23
NMO antibodies	137.55
Lupus anticoagulant	25.63
Anti-acetylcholine receptor antibodies	82.34
Triple tissue antibodies	20.84
BP 180 antibodies	58.36
BP 230 antibodies	58.36
Haptoglobin	10.38
Coombs test (direct)	10.49
Coombs test (indirect)	30.08
Coombs test	29.46
Total IgE	12.94
Anti-peroxidase Ac	10.42
Anti-ENA antibodies	31.66
Anti-transglutaminase antibodies	49.05
Cryoglobulins	19.21
Cryoagglutinins	21.55
Immunoglobulins	7.46
Anti DNA test	29.81
**Vitamins**	
Folic acid	16.63
Vitamin B12	18.37
Vitamin D	27.30
**Hormones**	
TSH	17.55
T3	11.21

T4	16.30
Intact PTH	37.59
ACTH	21.21
Thyroglobulin	24.94
Cortisol	16.37
Glycated hemoglobin	14.56
**Genetic study**	
TPMT	41.68
*HLA-B*27*	36.36
**Virus**	
Simple VH	24.00
Varicella zoster virus	34.47
Toxoplasma	31.06
Epstein-Barr virus test	20.32
Cytomegalovirus	21.07
HBV	50.80
HCV	36.27
HIV	161.54
Rubella	17.87
Influenza	55.88
**Histological study**	
Liver biopsy	395.20
Conjunctival biopsy	1304.69
Renal biopsy	239.73
Abdominal fat FNA	241.97
Colon biopsy	144.67
Skin biopsy FNA	62.36
Bone marrow biopsy with cytogenetic study	109.88
Gastric biopsy	388.17
**Cultures and microbiological studies**	
Antigens in urine	30.60
Urine biochemistry	12.46
Blood culture	21.39
Urine culture	10.01
Sputum culture	23.17
Stool culture	13.63
Stool parasites	15.19
Fecal occult blood	4.47
Catheter tip culture	9.65
Culture of superficial wound	20.62
Pharyngeal exudate and culture	14.62

Vaginal exudate and culture	14.94
Conjunctival exudate	20.00
CSF culture	20.79
Mantoux test	36.76
Clostridium difficile Toxin	25.88
Galactomannan Aspergillus	20.00
**Tumor markers**	
Alpha fetoprotein	12.59
Beta 2 microglobulin	15.00
carcinoembryonic antigen	12.17
Ca 199 antigen	16.37
Prostate specific antigen (PSA)	21.14
CA -125	12.14
CA-153	14.25
**Other**	
Peripheral blood immunophenotype	26.34
Peripheral blood smear	5.35
Immunofixation in serum	43.83
ProBNP	40.67
CK.CK(MB)	13.05
Electrocardiograms	24.84
Electroencephalogram	102.49
Electromyogram	123.28
Plethysmography	80.21
Spirometry	44.33
Nitric oxide determination	17.71
**Imaging studies**	
**X-rays**	
Chest X-ray	19.39
Abdominal X-ray	24.24
Cervical X-ray	21.16
Dorsal X-ray	23.46
Lumbar Spine X-Ray	20.05
Bone Region X-Ray	13.43
Hip X-Ray	20.81
Knee X-Ray	24.76
Elbow X-Ray	19.06
Ankle/Foot X-Ray	25.80
Orthopantography	32.44
Wrist X-Ray	18.75
Opaque Enema X-Ray	143.41

Intestinal transit radiography	218.59
Bone densitometry	73.72
Urography	211.84
Cystography	146.93
**Ultrasound**	
Thyroid ultrasound	54.44
Soft tissue ultrasound	63.33
Cervical ultrasound	58.99
Doppler ultrasound of lower limbs	142.30
Abdominal ultrasound	81.09
Gynecological ultrasound	54.74
Transthoracic Echocardiogram	105.03
**Tomography (CT) and Nuclear Magnetic Resonance Imaging (NMR)**	
Brain CT	135.75
Abdomen CT	135.00
Chest CT	124.49
Abdominal Thoracic CT	164.16
TACAR (High Resolution CT)	59.05
Cerebral angiography	1066.01
Facial and neck CT	145.75
Abdomino-pelvic CT	245.46
Brain MRI	265.18
Cervical MRI	280.47
Whole MRI	483.31
Dorsal MRI	255.88
Lumbar MRI	258.02
MRI cholangio	317.29
MRI lumbar dorsal	165.97
MRI Sacroiliac MRI	231.96
Lumbosacral MRI	128.00
MRI of the hip	214.51
Cerebral arteriography	642.46
Coronary angiography	359.63
Thyroid scan	43.25
**Procedures and other tests**	
Colonoscopy with biopsy	267.13
Gastroduodenoscopy	338.47
Rectoscopy	86.09
H pylori TEST	72.11
Gait test	192.29
Cystoscopy	180.77

Manometry	389.71
Psychology	70.35
Capillaroscopy	232.90
Respiratory Physiotherapy	12.24
Campimetry	95.36
**Hospital consultations by service**	
Rheumatology	112.47
Digestive	201.50
Hematology	110.15
Dermatology	74.31
Neurology	138.56
Internal Medicine	226.01
Pneumology	188.99
Nephrology	240.20
Ophthalmology	103.71
Traumatology	90.63
Maxillofacial Surgery	84.22
Nutrition	84.07
General surgery	133.17
Allergy	116.45
Neurosurgery	151.55
Cardiology	84.62
Endocrinology	139.82
Urology	97.80
Medical Oncology	137.26
Radiation Oncology	111.80
Otorhinolaryngology	52.96
Thoracic surgery	124.60
Vascular surgery	67.97
Rehabilitation	103.21
Day hospital	228.48
Cures	38.68
Nurse's office	18.31
Psychiatry	92.43
Anesthesia	57.95
Respiratory Physiotherapy	12.24
Neurophysiology	202.85
Infectious	162.76
**Admissions (average cost per day)**	
Emergency room	104.52
Urology	850.88

Nephrology	862.69
Rheumatology	923.76
Digestive	557.74
Intensive Care Unit	1752.37
Hematology	818.95
General and digestive surgery	808.15
Ophthalmology	1633.30
Pneumology	514.50
Cardiology	1024.12
Internal Medicine	478.57
Neurosurgery	960.95
Neurology	622.87
**Procedures**	
Pigtail placement	705.00
Red blood cell concentrates	92.00
Hemostasis study	7.69
Keratoplasty	476.50
Fibrobronchoscopy	147.19
Cholecystectomy surgery	2912.94
Cryotherapy	51.46
Lumbar puncture	243.16
Plaquetoaferesis	467.00
Caudal block	651.21
Central venous catheter	625.79
Operating room	558.68

Supplementary Table S2. Total healthcare cost per patient and breakdown by cost of tests, consultations, hospital admissions and cost of medication

Patient ID	Total cost of tests (€)	Total cost of consultations (€)	Cost of hospital admission (€)	Cost of thiopurine treatment (€)	Cost of concomitant treatment (€)	Total healthcare cost (€)
10	745.71	1088.90	5272.94	36.08	43.26	7186.89
20	395.41	806.01		275.40		1476.82
30	520.32	1012.23	209.05	36.08	30.00	1807.67
40	321.76	449.88		27.06	7.50	806.20
50	766.08	495.87	19943.75	45.10	11.28	21262.08
70	864.46	438.79	9618.13	27.06	17.50	10965.94
90	134.46	486.02		27.06	262.35	909.90
100	1190.38	1117.66	6632.66	36.08	392.14	9368.92
120	748.38	1082.49	104.52	99.22	11.70	2046.31
130	807.92	987.26	10872.65	18.04	11.28	12697.16
140	374.79	462.58		36.08	15.04	888.49
150	259.08	462.16		72.16	46.83	840.23
160	259.08	716.83		72.16		1048.07
170	329.18	562.35		135.30	23.04	1049.87
180	185.37	371.53		135.30	15.00	707.20
200	271.06	674.82	104.52	72.16	15.00	1137.56
230	469.74	1007.51	1219.99	117.26	15.00	2829.51
240	748.34	1410.52	104.52	54.12	392.14	2709.64
260	480.49	1410.52	104.52	81.18	4592.61	6669.32
270	185.37	537.42		72.16	20.00	814.95
290	195.84	297.22		72.16	15.00	580.22
300	159.92	371.53		72.16	15.00	618.60
310	620.29	1696.09		99.22	392.14	2807.73
320	671.88	2732.01	104.52	81.18	40.48	3630.07
330	156.67	297.22		135.30	15.00	604.19
340	2276.44	1007.51	16275.23	99.22		19658.41
380	77.60	74.31		9.02	20.00	180.93
390	113.52	148.61		90.20	20.00	372.34
400	1393.82	737.68	11804.44	54.12		13990.06
410	418.83	1146.07	1115.47	135.30	392.14	3207.81
420	626.37	1007.51		117.26	10.00	1761.14
430	231.76	1081.82		99.22		1412.80
440	2016.57	1325.47	104.52	18.04		3464.60
450	113.52	1740.96	4737.89	99.22	15.04	6706.63

460	939.59	412.79	313.57	18.04	67.56	1751.55
470	1091.83	2095.97		99.22	6765.42	10052.44
480	1668.84	411.72	104.52	63.14	25.00	2273.22
490	1579.98	1146.96	104.52	76.50	15.00	2922.97
500	1538.32	1117.66	6129.36	45.10	392.14	9222.58
510	323.00	1319.16	12184.97	76.50	11.70	13915.33
520	353.77	876.32		36.08	15.00	1281.17
530	159.92	779.39		36.08	15.00	990.39
540	195.84	1209.02		36.08	20.00	1460.93
580	347.97	1209.02		72.16		1629.14
590	366.42	498.73	104.52	72.16	15.00	1056.83
620	2377.39	806.01	571.52	99.22	30.00	3884.14
650	776.22	1321.49	104.52	168.30		2370.53
710	766.25	1972.87	8569.98	76.50	30.00	11415.60
720	447.42	1321.49	25329.41	81.18		27179.50
730	1069.16	1209.02		63.14	86.80	2428.12
740	591.09	1183.99		45.10	168.06	1988.24
770	246.64	939.18	1673.21	18.04	300.00	3177.07
780	235.30	2758.10		72.16	4592.61	7658.16
790	603.97	1612.02	209.05	99.22	11.70	2535.96
800	2693.17	411.91	571.52	72.16	15.00	3763.76
820	333.39	628.06	522.61	45.10	23.04	1552.20
830	272.01	1190.92		99.22	22.56	1584.71
860	589.89	892.79	27485.20	99.22	5.85	29072.95
870	773.59	734.08	104.52	54.12	16.94	1683.25
890	273.64	657.95		81.18		1012.77
900	425.27	1007.51		99.22	353.68	1885.69
910	1287.50	1209.02		81.18	6765.42	9343.12
920	134.46	1007.51		99.22		1241.20
940	123.99	222.92		54.12	170.82	571.85
950	123.99	1612.02		117.26	12528.84	14382.12
960	319.61	1119.98		81.18	10.12	1530.90
970	113.52	403.01		168.30		684.83
980	193.19	403.01	104.52	99.22		799.94
990	1264.20	806.01		45.10	15.00	2130.31
1000	845.26	403.01		117.26	265.26	1630.79
1010	185.26	487.23		117.26		789.75
1020	185.26	604.51		99.22	4592.61	5481.60
1050	194.28	604.51		153.34	392.14	1344.27
1060	134.46	870.85		117.26		1122.57
1080	134.46	604.51		117.26	34.71	890.94

1090	958.39	1072.35		81.18	170.82	2282.75
1100	278.05	604.51		135.30	11.70	1029.56
1110	562.21	403.01		117.26	265.26	1347.74
1130	1013.87	1209.02		168.30		2391.19
1140	603.11	514.80		72.16	16.94	1207.01
1150	481.28	816.35	104.52	99.22	392.14	1893.51
1160	445.35	1007.51	209.05	135.30	392.14	2189.35
1170	597.06	1410.52		135.30	11.70	2154.58
1180	491.14	1360.57		99.22	1.95	1952.88
1190	731.83	1995.33	4848.87	117.26	392.14	8085.43
1200	180.86	1760.70		81.18	15.00	2037.74
1210	609.25	1760.63		168.30	75.90	2614.08
1220	797.82	2230.64	5974.75	168.30	6765.42	15936.93
1230	180.86	604.51		117.26	16.94	919.56
1240	141.92	1342.19		117.26		1601.37
1250	134.46	1757.76		135.30	392.14	2419.67
1260	819.99	995.00	209.05	99.22		2123.26
1270	466.23	604.51		54.12	4592.61	5717.47
1280	419.84	737.68		135.30		1292.81
1300	1287.01	604.51		117.26		2008.78
1310	134.46	403.01		135.30	392.14	1064.91
1320	2047.28	403.01	5890.92	135.30	4592.61	13069.12
1330	227.25	780.99		135.30	69.44	1212.97
1340	161.32	1556.26	112.21	27.06		1856.85
1350	419.84	520.14		99.22	3.90	1043.10
1360	190.62	1981.03	4461.88	99.22	156.24	6888.99
1370	180.86	524.19		36.08	15.00	756.12
1380	273.64	785.76		99.22	392.14	1550.76
1390	631.23	1061.47	104.52	99.22	4592.61	6489.06
1400	215.85	1692.96		99.22	4592.61	6600.64
1410	416.40	934.95	418.09	117.26	84.03	1970.73
1420	185.26	1007.51		81.18		1273.96
1430	975.55	806.01	2335.46	45.10	69.44	4231.57
1440	1393.68	806.01		99.22		2298.91
1450	769.90	1848.14		81.18	69.44	2768.66
1460	1225.31	604.51		99.22	16.94	1945.98
1470	299.74	2134.19		117.26		2551.19
1480	205.69	2256.11		99.22	11.70	2572.73
1490	205.69	2525.95		99.22	4592.61	7423.47
1500	205.69	1146.07		99.22	392.14	1843.13
1510	205.69	604.51		117.26	392.14	1319.60

1520	159.30	806.01		81.18		1046.49
1530	230.53	1007.51		99.22		1337.26
1540	369.70	793.50		122.40	15.00	1300.60
1550	205.69	708.21		81.18	224.08	1219.17
1560	267.52	1007.51		275.40		1550.43
1580	159.30	554.24		72.16	8.70	794.40
1590	205.69	1037.78		72.16	16.94	1332.57
1600	442.29	448.93		72.16	11.34	974.71
1610	180.86	148.61		45.10	30.00	404.57
1620	180.86	716.98		168.30	15.00	1081.13
1640	1794.99	806.01	209.05	229.50	392.14	3431.68
1650	273.64	1140.68		117.26		1531.58
1660	522.63	604.51		99.22	224.08	1450.44
1670	227.25	604.51		168.30	75.90	1075.96
1680	400.66	720.78	13207.64	36.08	6.26	14371.41
1700	134.46	1007.51		81.18	16.94	1240.10
1710	1105.95	1054.39	104.52	72.16	6765.42	9102.44
1720	227.25	297.22		72.16	11.70	608.33
1730	812.15	872.90		81.18	11.70	1777.93
1740	546.26	371.53		135.30	11.70	1064.79
1750	227.25	1961.25		229.50	4592.61	7010.61
1760	204.66	806.01	104.52	135.30		1250.49
1770	280.39	604.51		135.30	6765.42	7785.62
1780	219.96	604.51		135.30	168.06	1127.83
1790	686.97	1007.51		81.18		1775.66
1820	2256.88	604.51	15912.97	117.26		18891.61
1830	281.40	1924.28		99.22	15.00	2319.90
1840	538.82	604.51	209.05	99.22	265.26	1716.85
1860	303.91	737.68		99.22		1140.81
1870	130.68	1335.15	3743.10	36.08	156.24	5401.25
1890	467.05	806.01	104.52	135.30	15.00	1527.88
1900	178.54	1767.52		72.16	30.00	2048.23
1910	180.86	972.43		99.22	11.70	1264.20
1920	422.10	224.94		54.12	11.70	712.86
1930	267.24	604.51		99.22		970.97
1950	333.75	222.92		72.16	5.85	634.67
1970	208.72	222.92		72.16	22.56	526.36
1990	178.54	787.29		36.08	22.56	1024.47
2000	264.60	904.04		72.16	11.70	1252.50
2010	152.01	1409.02		117.26	15.00	1693.29
2050	134.46	708.21		36.08	15.00	893.76

2080	855.66	222.92	3400.57	72.16	11.70	4563.01
2090	457.23	337.41	104.52	36.08	11.70	946.95
2100	401.35	500.04		99.22	23.76	1024.36
2120	198.40	148.61		36.08	7.50	390.59
2130	465.55	377.98	2162.52	45.10	15.00	3066.15
2160	88.07	972.04		162.36	15.00	1237.47
2180	385.55	789.89		27.06	30.00	1232.50
2190	398.61	222.92		99.22	15.00	735.75
2210	258.38	188.99	5145.00	27.06	22.56	5641.99
2220	508.36	112.47	209.05	27.06	15.00	871.94
2230	367.05	744.98		72.16	30.00	1214.19
2240	341.64	371.53	104.52	108.24	12.50	938.43
2280	239.49	831.36		54.12	22.56	1147.53
2290	749.73	1220.91		72.16	5.85	2048.65
2310	624.96	371.53		135.30	11.28	1143.07
2330	227.25	429.19		54.12	11.70	722.25
2340	358.76	188.99	104.52	9.02	15.00	676.29
2350	828.21	944.95		72.16	15.00	1860.32
2360	518.32	1018.99		72.16	15.00	1624.47
2370	930.53	566.97	209.05	72.16	2.56	1781.26
2400	976.08	623.75	1133.52	9.02	10.04	2752.41
2410	134.46	933.97		81.18	15.00	1164.62
2420	424.60	447.56		27.06	15.00	914.22
2450	112.31	1234.13		135.30	1817.74	3299.48
2460	259.62	1372.76	4263.19	18.04	15.00	5928.61
2490	227.25	207.30		9.02	11.70	455.27
2500	463.77	889.13	104.52	36.08	30.00	1523.50
2510	1957.36	112.47	2997.72	9.02	15.00	5091.57
2520	320.59	371.53	104.52	99.22	85.41	981.27
2530	706.04	387.85		36.08	30.00	1159.97
2540	299.82	462.60	209.05	9.02	30.00	1010.48
2570	1481.20	380.83	8037.08	9.02	11.28	9919.40
2580	541.81	602.92		72.16	22.56	1239.45
2600	301.90	1141.83		81.18	23.04	1547.96
2610	1054.11	186.78	1133.52	27.06	30.00	2431.47
2630	388.05	892.79	9605.61	27.06	11.70	10925.21
2660	367.47	495.23	104.52	99.22	34.51	1100.95
2670	96.51	598.97		99.22	23.04	817.74
2690	635.36	767.89	104.52	72.16	15.00	1594.93
2710	213.29	1374.50		72.16	11.70	1671.66
2720	1605.79	459.43		99.22	23.04	2187.48

2730	221.54	112.47		9.02	15.00	358.03
2740	346.30	1557.03		72.16	11.70	1987.19
2760	233.01	647.34		135.30	15.00	1030.65
2770	137.27	1298.62	102.92	36.08	11.70	1586.60
2780	134.46	566.97		36.08	30.00	767.51
2790	604.95	566.97		81.18	30.00	1283.10
2810	88.07	653.68		99.22	15.00	855.97
2820	336.68	337.41		9.02	23.04	706.15
2830	383.08	355.72		9.02	23.04	770.86
2840	675.42	1156.30		72.16	22.56	1926.44
2850	264.52	371.53		72.16	7.50	715.71
2860	307.46	224.94		27.06	15.00	574.46
2880	796.11	445.83	104.52	135.30	15.00	1496.77
2890	349.69	755.96		81.18	15.00	1201.83
2900	180.86	345.97	104.52	18.04	1.95	651.34
2910	408.60	603.59	209.05	72.16	15.00	1308.39
2940	497.78	1037.67	8192.92	72.16	30.00	9830.53
2950	290.87	1098.37	104.52	72.16	15.00	1580.92
2960	447.35	222.92		54.12	170.82	895.20
2970	437.15	675.20		72.16		1184.51
2980	41.68	337.41		18.04	15.00	412.13
2990	88.07	470.83	1029.00	9.02	15.00	1611.92
3000	695.85	447.56		81.18	11.70	1236.29
3010	329.47	148.61	104.52	72.16	5.85	660.61
3020	409.45	130.78	209.05	18.04	15.00	782.32
3030	312.70	789.74		99.22	28.44	1230.11
3040	573.00	1016.97		72.16	15.00	1677.13
3050	1191.84	371.53		72.16	15.00	1650.53
3060	88.07	239.24		18.04	5.85	351.20
3080	458.93	1497.23		99.22	11.28	2066.65
3090	695.14	1764.45	313.57	72.16		2845.31
3100	450.15	1256.61		135.30	7.50	1849.56
3120	88.07	130.78		9.02	23.04	250.91
3150	341.27	337.41		90.20	30.00	798.88
3190	351.93	1452.17		36.08	12.52	1852.70
3200	730.53	1059.84		72.16	3.90	1866.43
3210	512.24	252.32		9.02	85.41	858.98
3220	116.51	749.51		99.22	15.00	980.24
3230	480.24	337.41	1209.89	72.16	145.92	2245.61
3250	425.14	112.47		9.02	11.70	558.33
3260	569.83	1213.48		72.16	30.00	1885.47

3270	785.72	941.99	104.52	99.22	15.00	1946.45
3280	480.65	703.33		135.30		1319.28
3310	3458.51	7997.36	2807.97	72.16	15.00	14351.00
3320	1166.31	835.36	104.52	72.16	11.70	2190.05
3330	113.87	1455.07		81.18	15.00	1665.12
3340	299.82	1597.62		99.22	15.00	2011.65
3350	189.30	1068.73	243.16	27.06	15.00	1543.25
3380	134.46	148.61	3349.99	18.04	10.00	3661.11
3410	488.06	138.56	418.09	9.02	30.00	1083.73
3420	206.31	602.14		99.22	18.80	926.47
3430	168.28	307.54		99.22	2.50	577.54
3440	88.07	465.66		81.18	2.50	637.41
3460	501.81	902.43	104.52	72.16	15.00	1595.91
3470	365.35	243.25		27.06	15.00	650.66
3480	603.83	207.30		9.02	30.00	850.15
3490	615.13	188.99		18.04	15.00	837.16
3500	308.34	461.98	9301.88	18.04	15.04	10105.27
3510	605.99	1000.27	209.05	99.22	156.24	2070.76

(a)

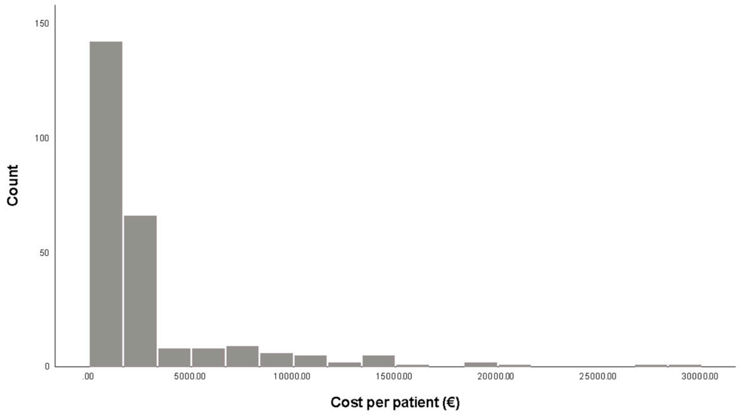

(b)

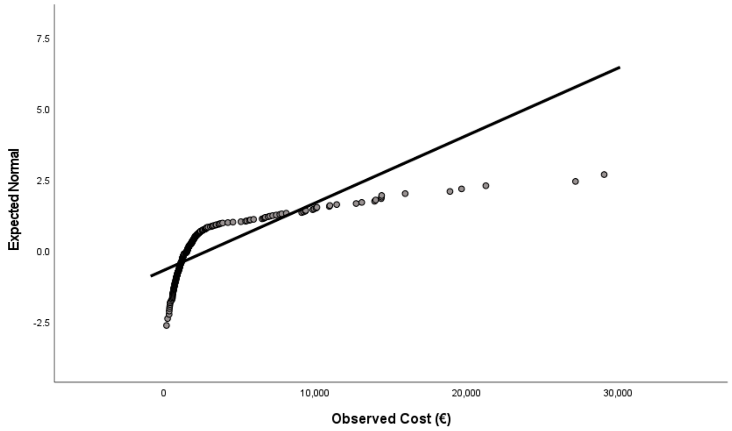

Supplementary Figure S1. Normality plots for the “Cost per patient (€)” variable. (a) Histogram showing the asymmetrical, leptokurtic distribution of the variable, (b) Quantile-quantile plot of the observed “Cost per patient (€)” value and the expected normal value.
